# Toxic metals in cord blood and later development of Type 1 diabetes

**DOI:** 10.15761/PD.1000186

**Published:** 2019-05-24

**Authors:** J Ludvigsson, P Andersson-White, C Guerrero-Bosagna

**Affiliations:** 1 Department of Clinical and Experimental Medicine, Division of Pediatrics, Linköping University, Linköping, Sweden; 2 Crown Princess Victoria Children’s Hospital, Region Östergötland. Linköping Sweden; 3 IFM Biology, Linköping, University; Linköping university, Linköping, Sweden

**Keywords:** type 1 diabetes, toxic metals, aetiology, ABIS, children

## Abstract

The incidence of type 1 diabetes (T1D) has increased explained by changes in environment or lifestyle. In modern society dissemination of heavy metals has increased. As the autoimmune process usually starts already, we hypothesized that exposure to toxic metals during fetal life might contribute to development of T1D in children.

We analysed arsenic (AS), aluminium (Al), cadmium (Cd), lithium (Li), mercury (Hg), lead (Pb), in cord blood of 20 children who later developed T1D (probands), and in 40 age-and sex-matched controls. Analysis of heavy metals in cord blood was performed by ALS Scandinavia AB (Luleå, Sweden) using the ‘ultrasensitive inductively coupled plasma sector field mass spectrometry method’ (ICP-SFMS) after acid digestion with HNO_3_.

Most children had no increased concentrations of the metals in cord blood. However, children who later developed T1D had more often increased concentrations (above limit of detection; LOD) of aluminium (p = 0.006) in cord blood than the non-diabetic controls, and also more often mercury and arsenic (n.s).

Our conclusion is that exposure to toxic metals during pregnancy might be one among several contributing environmental factors to the disease process if confirmed in other birth cohort trials.

## Introduction

The incidence of type 1 diabetes (T1D) has increased over decades. This must be explained by changes in environment or lifestyle, but the aetiology is unknown [1]. As the autoimmune process often starts very early in life the pathogenic factors might be found in fetal life. In modern society dissemination of heavy metals and human exposure has increased, which has been associated to T1D. Some minerals which part of the normal physiology may play a role. Thus, it is known that iron may be toxic for beta cells and studies suggest that increased iron consumption in nutrients may be associated with increased risk of T1D [2]. It has also been found that high iron concentrations at birth is associated with an increased risk of T1D [3]. Deficiency of zinc, a central part of the insulin hexamer, is regarded to be related to increased risk of diabetes and there are also epidemiological studies supporting a role for low zinc in the risk for T1D [4] while low perinatal zinc status was not associated with the risk of T1D later in children [5]. However, metals that have no physiological role in the human physiology but should rather be regarded as toxic, may play a role. Thus arsenic, cadmium, mercury and nickel have been found to have toxic effect on beta cell function [6], and cadmium concentration to be higher in urine of prediabetic and diabetic patients than of controls [7]. In animal studies cadmium may cause beta cell destruction. High concentration of nitrate as well as mercury and arsenic in drinking water has been suspected to increase the risk of developing type 1 diabetes [8]. Thus, heavy metals in foods or water might influence the autoimmune mechanisms in genetically susceptible individuals, and exposure to toxins might result in pancreatic islet cell death.

With this background, and the fact that the autoimmune process usually starts already in infants we hypothesized that exposure to toxic heavy metals during fetal life might in some way contribute to later development of that process which leads to T1D in children.

## Material and methods

ABIS (All Babies in Southeast Sweden) is a cohort trial with children born Oct 1^st^ 1997-Oct 1^st^ 1999 followed prospectively from birth with the aim to study causes of immune-mediated diseases in a general population [9]. We chose randomly 20 children (probands) among those who have developed T1D and in addition 40 age and sex-matched controls, who have remained non-diabetic. Parents or guardians gave their informed consent and the protocol of the ABIS study was approved by the Research Ethics Committees of the Faculty of Health Sciences, Linköping University, and the Medical Faculty, Lund University, Sweden 1997–02-19 Dnr 96287.

The following metals were analysed in cord blood of 20 probands and in 40 controls (arsenic only in 10 controls): arsenic (AS), aluminium (Al), cadmium (Cd), lithium (Li), mercury (Hg), lead (Pb). Analysis of heavy metals in cord blood was performed by ALS Scandinavia AB (Lulea, Sweden) using the ‘ultrasensitive inductively coupled plasma sector field mass spectrometry method’ (ICP-SFMS) after acid digestion with HNO_3_, according to the standards ISO 17294–1 (https://www.iso.org/standard/32957.html) and 2 (https://www.iso.org/standard/62962.html), and the EPA Method 200.8 (https://www.epa.gov/homelandsecurity-research/epa-method-2008-determination-trace-elementswaters-and-wastes) [10].

### Statistical analysis

Data were found to be left censored for all metals with a large percentage of cases with values below the limit of detection (LOD) making analysis with parametric test unsuitable. Instead the distribution of values above or below LOD for respective metal was compared and tested using Chi-square test of independence or Fischer’s exact test when cell count was found to be below 5. Data was analyzed with SPSS version 23.0.

## Results

**Figure 1.**
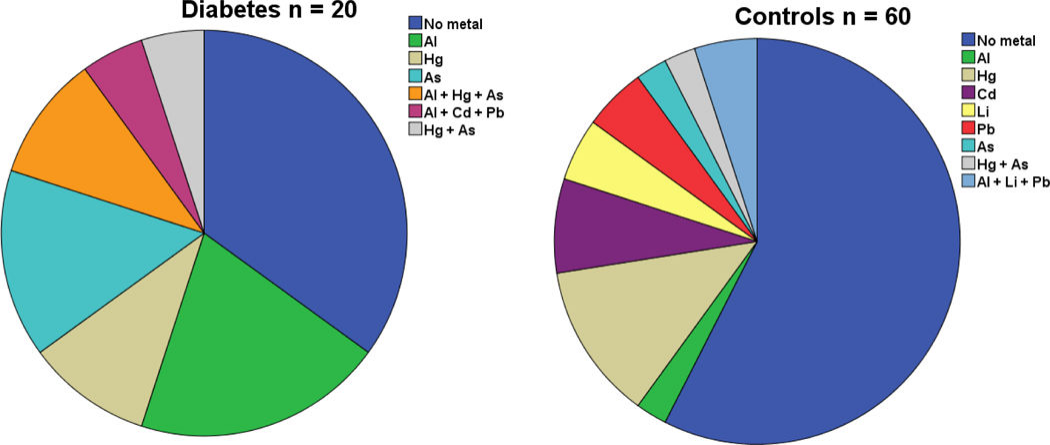
Children who later developed Type 1 diabetes (n=20) have more often one or more toxic metals detectable in their cord blood than what is found in cord-blood of control children, born very close in time, who remain non-diabetic.

Concentrations of the metals in cord blood in the probands and the controls are seen in Table 1. Most of the children, both probands and controls, had no increased concentrations of the metals in cord blood. However, children who later developed T1D had significantly more often increased concentrations (above limit of detection; LOD) of aluminium in cord blood than the non-diabetic controls (*p* = 0.006). There was no significant difference between the groups for any other single metal, but both mercury and arsenic were more common (n.s) in the probands (Figure 1). Those who developed T1D later had significantly more often increased concentrations of the combination of aluminium and arsenic.

## Discussion

In a prospective study with water quality measured before the onset of islet autoimmunity and T1D, concentrations of nitrate, nitrite, iron, aluminium, and manganese were not found to be associated with risk of T1D progression [11]. However, as mentioned above there are indications that heavy non-physiological metals may have toxic effects on beta cells [6] and exposure to such metals has been suspected to increase the risk of developing type 1 diabetes [8] but we know of no published studies where exposure to toxic metals during pregnancy has been analyzed.

Here we found that aluminium more often was increased above the level of detection in cord blood of children who later develop T1D. Aluminium has to our knowledge not been previously associated to T1D but been discussed as a possible risk factor for neurological diseases such as autism [11]. We also found mercury and arsenic slightly more often in cord blood of children who later develop T1D. Mercury is supposed to influence the immune system and could contribute to the development of autoimmune diseases [12]. The cells and organ systems can be supposed to be more sensitive to toxic agents during the very early fetal life, and therefore our findings is an observation that should be studied further.

A limitation of our study is of course the low numbers. This might explain that some differences seen between exposure to mercury and arsenic do not become statistically significant, but it is then even more remarkable that we see statistically significant differences in prevalence of toxic metals in cord blood. As several individuals have a high number of measures below the limit of detection a more correct statistical approach to use might have been a zero-inflated model, but our numbers do not allow this.

In conclusion, in most of the cord blood samples in children who later develop T1D we do not find any increase of toxic metals and such exposure therefore does not seem to be of common or heavy importance. It is necessary to be cautious drawing conclusions from our small material, but we find more often increased concentrations of aluminium and, although non-significantly, also more often mercury and arsenic in cord blood of children who later develop T1D compared to what is found in nondiabetic controls. These findings suggest that exposure to toxic metals during pregnancy might be one among several contributing environmental factors to the disease process if confirmed in other studies [13].

## Figures and Tables

**Table 1.** Distribution of metal concentration above the limit of detection in cord blood at birth in diabetic patients and controls. Limit of detection for each metal; Aluminium 7.0 μg/l, Cadmium 0.07 μg/l, Lithium 1.0 μg/l, Mercury 0.3 μg/l, Lead 0.7 μg/l, Arsenic 1.0 μg/l.

	Controls (%)	Type 1 Diabetes (%)	Total (%)	*p*
Aluminium (Al)	2 (5.4%)	7 (35.0%)	9 (15.8 %)	0.006^a^
Cadmium (Cd)	5 (12.5%)	1 (5.0%)	6 (10.0%)	0.653^a^
Lithium (Li)	3 (7.7%)	0 (0.0%)	3 (5.1%)	0.544^a^
Mercury (Hg)	5 (12.5%)	5 (25%)	10 (16.7%)	0.278^a^
Lead (Pb)	5 (12.5%)	1 (5.0%)	6 (10.0%)	0.653^a^
Arsenic (As)	1 (10.0%)	6 (30%)	7 (23.3%)	0.372 ^a^
Any metal*	16 (43.2%	10 (50.0%)	26 (45.6%)	0.625^b^
Any ≥ 2 metals*	3 (8.1%)	3 (15.0%)	6 (10.5%)	0.654^a^
Any 3 metals*	1 (2.7%)	1 (5.0%)	2 (3.5%)	1.00^a^
Any metal**	17 (45.9%	13 (65.0%)	30 (52.6%)	0.169 ^b^
Any ≥ 2 metals**	3 (8.1%)	4 (20.0%)	7 (12.3%)	0.226 ^a^
Any 3 metals**	1 (2.7%)	3 (15.0%)	4 (7.0%)	0.119 ^a^
Aland Hg	0 (0%)	2 (10%)	2 (3.5 %)	0.119^a^
As and Hg	0 (0.0%)	3 (15.0%)	3 (5.0%)	0.033 ^a^
Cd and Pb	2 (5%)	1 (5%)	3 (5%)	1.00

a Fischer’s Exact test

b Chi-square test

* Arsenic not included

** Including known values for Arsenic. Missing values for Arsenic are assumed to be below detection
